# A Smartphone App to Monitor Mood Symptoms in Bipolar Disorder: Development and Usability Study

**DOI:** 10.2196/19476

**Published:** 2020-09-22

**Authors:** Kelly Ann Ryan, Pallavi Babu, Rebecca Easter, Erika Saunders, Andy Jinseok Lee, Predrag Klasnja, Lilia Verchinina, Valerie Micol, Brent Doil, Melvin G McInnis, Amy M Kilbourne

**Affiliations:** 1 Department of Psychiatry University of Michigan Ann Arbor, MI United States; 2 Department of Psychiatry Pennsylvania State University Hershey, PA United States; 3 School of Information University of Michigan Ann Arbor, MI United States; 4 Brehm Center for Diabetes Research University of Michigan Ann Arbor, MI United States; 5 Department of Learning Health Sciences University of Michigan Ann Arbor, MI United States; 6 US Department of Veterans Affairs Health Services Research & Development VA Depart of Veterans Affairs Ann Arbor, MI United States

**Keywords:** bipolar disorder, momentary assessment, mood, mobile phone, mobile app

## Abstract

**Background:**

There is considerable scientific interest in finding new and innovative ways to capture rapid fluctuations in functioning within individuals with bipolar disorder (BD), a severe, recurrent mental disorder associated with frequent shifts in symptoms and functioning. The use of smartphones can provide valid and real-world tools for use in measurement-based care and could be used to inform more personalized treatment options for this group, which can improve standard of care.

**Objective:**

We examined the feasibility and usability of a smartphone to capture daily fluctuations in mood within BD and to relate daily self-rated mood to smartphone use behaviors indicative of psychomotor activity or symptoms of the illness.

**Methods:**

Participants were 26 individuals with BD and 12 healthy control individuals who were recruited from the Prechter Longitudinal Study of BD. All were given a smartphone with a custom-built app and prompted twice a day to complete questions of mood for 28 days. The app automatically and unobtrusively collected phone usage data. A poststudy satisfaction survey was also completed.

**Results:**

Our sample showed a very high adherence rate to the daily momentary assessments (91% of the 58 prompts completed). Multivariate mixed effect models showed that an increase in rapid thoughts over time was associated with a decrease in outgoing text messages (β=–.02; *P*=.04), and an increase in impulsivity self-ratings was related to a decrease in total call duration (β=–.29; *P*=.02). Participants generally reported positive experiences using the smartphone and completing daily prompts.

**Conclusions:**

Use of mobile technology shows promise as a way to collect important clinical information that can be used to inform treatment decision making and monitor outcomes in a manner that is not overly burdensome to the patient or providers, highlighting its potential use in measurement-based care.

## Introduction

Bipolar disorder (BD) is a severe, recurrent mental disorder that often has devastating effects on patient’s everyday functioning. BD is characterized by intermittent episodes of depression and mania or hypomania, and it affects at least 2% of the general population, yet it is the sixth leading cause of disability worldwide in individuals aged 18 to 44 years [1] and the most costly mental health condition [2]. Taken together, this frequently shifting illness leads to substantial comorbidity, health care costs, and premature mortality [3,4]. Further, many patients with BD experience significant daily and weekly mood swings that are below the threshold for a full mood episode. It is typically this mood instability that can contribute to impairments in daily functioning [5]. Among those who seek or receive treatment, many do so within primary care [6-8], where precision in patient monitoring of problematic functioning is time and resource consuming.

Provider-administered assessments used in research or in clinical settings can be difficult to implement in routine clinical practice due to lack of clinical psychiatric training and time constraints, leading to the growing use of self-completed symptoms assessments. However, self-completed assessments of mood symptoms are dependent on retrospective reporting, which typically asks about symptoms experienced over the past week or two, which is subject to recall and response bias. Such assessments also require aggregation of mood-specific symptoms over days by the patient and the clinician, which is difficult to sustain in real-world clinical settings. Therefore, there is a need for more practical assessment of rapid fluctuations and symptoms in mood within BD.

In order to capture rapid fluctuations in functioning within individuals with BD, a more clinically useful measurement is needed to inform more personalized treatment options for this group and improve standard of care. The use of measurement-based care, a practice of basing clinical care on patient data that are continuously collected to inform treatment decision making and monitor outcomes [9], has not been widely adopted in mental health treatment due to the burden of assessments in routine practice. However, the advent of measurement-based care and precision medicine has created a potential for the development of valid and reliable methods to measure individual variation in symptoms and functioning, which could better inform treatments through improved diagnosis, predicted relapse, and insight into disease progression.

The use of mobile health technologies or remote measurement technology (ie, smartphone and similar technologies) has immense promise to not only capture reported symptom information but also provide data on phone behaviors (eg, information about number of phone calls or text messages being sent or received) that could indicate changes in functioning, both of which are ecologically valid ways of capturing behaviors in the real world [10,11]. Additionally, mobile technology offers a delivery method for assessment of what people are doing in real-world settings, a methodology known as ecological momentary assessment (EMA) [12]. Using EMA on smartphones can also provide a real-time clinical management tool for psychiatric illness by providing an early warning sign of problematic shifts in mood or changes in behavior. EMA techniques are advantageous over retrospective reports, can be collected in the moment, and can be coupled with data on other smartphone usage behaviors to provide a unique way of accurately and efficiently monitoring changes in functioning and clinical phenotypes. Further, mobile technology can serve as a way in which patients can self-monitor their symptoms and functioning, which subsequently can allow them to take part in the medical decision making. Mobile technology can provide a quick and cost-efficient way to inform providers that patients are in the beginning stages of a mood episode relapse. Using mobile technology in this way can further push forward the feasibility of measurement-based care and help prevent disease progression, relapse, and treatment costs; however, this has not been rigorously tested.

There has been a surge of research examining the use of smartphones to monitor and capture symptoms and behaviors across a range of illnesses, including psychiatric illness [13-17], suggesting that there is considerable scientific interest in finding new and innovative ways of measuring illness parameters. Recent studies show mobile electronic devices to be useful in assessing and monitoring behaviors and severity of symptoms in both inpatient and outpatient settings [18,19]. Other studies have used mobile phone technologies to determine a relationship between psychiatric symptoms and phone behavior [20-22]. For example, Faurholt-Jepsen and colleagues [21] found that more depressive symptoms were associated with the phone’s screen being on for longer periods, more incoming calls, fewer answered incoming calls, and less movement from the cell towers. In contrast, more manic symptoms were associated with more outgoing text messages and longer phone calls, suggesting that smartphones can generate objective data for behavioral activities that may be markers of illness activity.

Further examination of the feasibility and acceptance of using EMA on smartphones among those with BD illness is crucial. In addition, understanding if momentary assessment of mood is related to phone usage data, such as phone calls and text messages sent or received, is needed if the goal is to leverage technology for measurement-based care in a range of clinical settings. The aims of this study were (1) to examine the feasibility and acceptability of using a smartphone to capture daily mood symptoms in BD, using a custom, clinically informed smartphone app, and (2) to relate daily self-rated mood to smartphone use behaviors, such as phone calls and text messages, that may be proxies for psychomotor activity or symptoms of the illness. We hypothesized that patients with BD will find using smartphones a feasible and useful way of monitoring symptoms, based on metrics of how compliant they were with completing daily tasks. Further, we hypothesized that phone usage data would be related to aspects of self-reported mood collected over 28 days. Specifically, we hypothesized that higher depression scores would be negatively correlated with number of phone calls and text messages made and time spent on phone calls, based on the psychomotor retardation and anhedonia often accompanying depression, and increased activity during mania would be positively correlated with number of phone calls, text messages, and time spent on phone calls [23,24].

## Methods

### Recruitment

Participants were recruited from the Heinz C Prechter Longitudinal Study of BD, an observational, naturalistic cohort study gathering phenotypic and biological data, at the University of Michigan [25]. A total of 36 individuals from the Prechter sample were enrolled into this current study (see “Results” section for breakdown). Recruitment and initial baseline evaluation procedures for the Prechter Longitudinal Study of BD have been described elsewhere [25], but to briefly summarize, all participants underwent an evaluation using the Diagnostic Interview for Genetic Studies (DIGS) [26], and a best-estimate process by at least two of the authors was used to confirm diagnoses. Participants were excluded if they had active substance use or neurological disease at enrollment. Both the longitudinal study and the present study were approved by the University of Michigan Institutional Review Board, and all participants provided signed informed consent.

Clinical information, such as age of onset for BD illness (DIGS), history of rapid cycling (based on having a history of 4 or more mood episodes per year) (DIGS), depression symptoms (Hamilton Depression Rating Scale-17 item) [27], mania symptoms (Young Mania Rating Scale) [28], and IQ (Wechsler Abbreviated Scale of Intelligence [29]), was taken from the initial baseline evaluation during enrollment into the larger longitudinal study. The number of lifetime mood episodes and psychiatric hospitalizations were obtained from the longitudinal data (DIGS) available in the Prechter Longitudinal Study. Inclusion criteria for the group with BD for this study included a history of rapid cycling or personal experience of mood fluctuations over the last couple months. Both BD and healthy control (HC) participants were included if they were currently smartphone users; all were provided with a study-specific smartphone. A total of 17 participants used iPhones at the time of enrollment but identified as being familiar with and having prior use with the Android operating system. We also selected participants who used their phones “regularly” and who were the primary users of their personal smartphones. Participants were excluded if they had vision or motor problems that would preclude them from using the smartphone study device or if they were not the primary or sole users of their smartphones (n=3). Participants received monetary compensation for their time participating in this study. They received US $160 at the end of the first 2 weeks of cell phone data collection, regardless of how many cell phone engagements they chose to use, and US $160 at the end of the study when they returned the issued smartphone.

### Procedure

#### Description of Smartphone App

The smartphone app was custom built to use Android 5.1.1 operating system, downloaded by invitation from Google Play. Data were stored securely on the Google App Engine. No personal or identifiable information was stored on the App Engine. The app was run continuously in the background of the phone with no indication to the participant that it was automatically collecting information about number and duration of phone calls and text messages. The app used minimal battery so battery drain was not readily apparent to the participant. Daily prompts to complete the app questions were sent in the morning and in the evening based on preselected times selected by the participants during study setup. They knew when these prompts would occur and they were prompted to complete app questions every 15 minutes for an hour until completed. No further prompts were sent after 1 hour. Notification of the prompts was visible by the study icon appearing at the top of the phone and accompanied by a unique prompting tone and message. Participants could swipe the screen message to launch the daily questions or delay for up to an hour. Participants responded to each question by using the visual analog scale ranging from 0 to 100 by positioning a marker on the touch screen (for mood questions, see “Smartphone Assessment” section below). The middle mark of 50 was the default position. During the training, participants were instructed that 0 and 100 represented the most extreme states they could imagine experiencing for each item and were provided examples to illustrate these points (ie, 0 on the impulsivity question was described as being the least impulsive in behaviors, so not reacting at all before thinking, and 100 was described as definitely acting or reacting without any forethought). After each response, participants pressed “Next” to continue to the next question. Time to complete the morning app questions was less than 5 minutes. Once they completed their session, the app responded with “Thank you for completing the survey” and automatically closed until the next scheduled prompt.

The app continuously and automatically collected phone usage information every day on (1) number of phone calls sent and received, (2) number of text messages sent and received, and (3) time spent on phone calls. The phones were set up to use wireless connectivity; data went to secure servers that could be checked by researchers at any time. No actual content within text messages was captured in order to maintain privacy.

#### Smartphone Assessment

At chosen prompt times in the morning and in the evening, participants answered 5 questions about their mood at that moment using a visual analog scale on days 1 to 14 and 28 to 56, for a total of 28 days. The rationale for the delivery of the daily prompts for 2 weeks with a 2 week gap was to decrease participant burden across 2 months but also to increase the chances that we collected some dynamic shifts in mood that may have occurred. These questions have been previously validated in a BD sample using a smartphone EMA study [30]. The 5 questions were (1) Rate your mood right now; (2) Rate your energy right now; (3) How fast are your thoughts right now? (4) How impulsive are your thoughts right now? and (5) How impulsive are your actions right now? These measures of symptomatology were selected based on correspondence to independent factors of mania shown through factor analysis: dysphoric mood (mood), psychomotor pressure (energy), psychosis (not conducive to self-report measure), increased hedonic function (impulsivity), and irritable aggression [31,32]. Further, mood, energy, and intellect (thoughts) were the 3 areas noted through careful longitudinal study of phenomenology by Kraepelin [33] to cycle out of sync, producing mixed states [34].

#### Prestudy Assessment and Device Training and Postassessment

After consenting, participants completed a 15-minute training session led by trained research associates reviewing the use of an Android smartphone and learning how to respond to the daily app questions. Responses to the daily questions were demonstrated and participants were given an opportunity to practice the use of the responses and ask questions as needed.

All participants were given a Samsung Galaxy Note 4 (Samsung Electronics) smartphone running with the Android 5.1.1 operating system, a charger, and an abbreviated user manual (with a link to a full phone manual). The device was matched to the participant’s personal cell phone carrier and their SIM card was placed into the study phone. Two participants chose not to use their current phone services (and personal cell phone numbers) and were provided with study phones with cellular data packages and new phone numbers. One participant did not participate because their personal SIM card was not compatible with the study phone and the participant did not want a different phone number. The device provided to each participant was preinstalled with the smartphone app. Participants’ personal contacts, text messages, apps, photos, videos, and music were transferred to the study phones using the Wondershare program (Wondershare Software Co) [35]. Participants were instructed to respond to each of the app’s twice-daily prompts to complete questions but to otherwise use the device as they normally would in their daily lives.

All individuals were reassessed at the end of 56 days of using the smartphone. This postassessment included completing a poststudy questionnaire that asked about their experiences and satisfaction using the smartphone, participating in the study, and responding to the daily prompts. Frequencies from this poststudy questionnaire were used as the main dependent variable in the feasibility and acceptability analyses.

### Statistical Analyses

We used 2-tailed *t* tests and chi-square tests to describe demographic differences in our diagnostic groups. Bivariate correlations were used to describe the relationship between our adherence rates/app usage, demographics, IQ, clinical scales, and other descriptive variables. Frequency distributions were used to describe the outcomes from the postevaluation assessment results.

When examining the data collected from the smartphone app collapsed across both EMA collection periods, we conducted paired *t* tests to compare the 5 daily self-reported morning mood scores across 28 days—(1) Rate your mood right now; (2) Rate your energy right now; (3) How fast are your thoughts right now? (4) How impulsive are your thoughts right now? (5) How impulsive are your actions right now?—with the same 5 daily self-reported evening mood scores to examine acceptability of averaging these scores by day. These results showed no statistically significant differences between morning mood scores and evening mood scores for all 5 mood questions (all *P*>.05), indicating that each morning and evening mood question response was similar to combine into a single mood question mean score, creating 5 aggregated daily mood scores. We then used mixed effects linear regression models with random intercept to examine the associations of measurements of daily (1) number of phone calls sent and received, (2) number of text messages sent and received, and (3) time spent on phone calls with the 5 average daily mood scores over time (see 5 mood questions above) as outcomes, which took into account the clustering effect of multiple measures over the same participants. Average daily mood scores were used as predictors for phone patterns. All these models were run using just the BD sample adjusted for age, sex, and bipolar diagnosis type (ie, BD-I vs BD-II).

## Results

### Participant Characteristics

A total of 26 participants in this study had BD (BD-I=17, BD-II=8, BD not otherwise specified=1) and 12 were HC participants with no personal or family history of any psychiatric illness. Out of the 38 participants enrolled, 2 individuals did not complete postassessment measures due to personal or health reasons but completed the daily app prompts. One person was removed from the study due to losing the study phone twice. Due to technical problems with the prompts not appearing during the second survey period, 9 participants have some missing data points.

Descriptive statistics for participants by diagnostic group are in Table 1. The mean age of participants was 44.9 years (SD 9.0). There were no significant differences between the BD and HC participants in age (t_36_=–0.44; *P*=.66), education (t_36_=0.47; *P*=.64), or IQ (t_36_=–0.04; *P*=.97). The majority of our participants were female (27/38, 71%), with no difference in gender breakdown between BD and HC groups (*χ*^2^_1_=0.2; *P*=.71).

**Table 1:** Demographics of participants.

Characteristic		BD^a^ (n=26)	HC^b^ (n=12)	Chi-square (*df*)	*t* test (*df*)	*P* value
Age (years), mean (SD)		46.46 (10.55)	44.92 (9.04)	N/A	–0.438 (36)	.66
Gender, n (%)		19 (73)	8 (67)	0.2 (1)	N/A	.69
Years of education, mean (SD)		15.88 (1.86)	16.17 (1.34)	N/A	0.470 (36)	.64
WASI IQ^c^, mean (SD)		108.96 (7.86)	108.83 (11.41)	N/A	–0.040 (36)	.97
HRDS^d^, mean (SD)		12.65 (6.49)	1.25 (1.87)	N/A	–5.933 (36)	<.001
YMRS^e^, mean (SD)		4.50 (4.92)	0.08 (0.29)	N/A	–3.086 (36)	.004
**Clinical features for the BD group**						
	Type of BD (BD-I/BD-II/BD-NOS^f^), n	17/8/1	N/A^g^	N/A	N/A	N/A
	Rapid cycling (yes), n (%)	15 (58)	N/A	N/A	N/A	N/A
	Age of BD onset, mean (SD)	16.31 (8.06)	N/A	N/A	N/A	N/A
	Compliance for full study (%), mean (SD)	92.20 (6.64)	90.63 (9.59)	N/A	–0.535 (31)	.60
	Compliance for first 2 weeks of study (%), mean (SD)	92.32 (9.89)	91.83 (8.61)	N/A	–0.147 (36)	.88

^a^BD: bipolar disorder.

^b^HC: health control.

^c^WASI IQ: Wechsler Abbreviated Scale of Intelligence.

^d^HRDS: Hamilton Rating Scale of Depression.

^e^YMRS: Young Mania Rating Scale.

^f^NOS: not otherwise specified.

^g^N/A: not applicable.

### Study Adherence

Overall, our sample completed 91.5% of prompts sent. There was no difference in total completion rate between the BD and HC groups (t_31_=–0.80; *P*=.43). Bivariate correlations (Table 2) showed that full study adherence was positively related to IQ (*r*=0.45; *P*=.008), with those with higher IQ completing more app question prompts. However, no other demographics or clinical variables were related to adherence.

**Table 2:** Correlations between compliance rates, demographics, and mood ratings.

Variable						Compliance first^a^		Compliance total^b^		Age	Education	WASI IQ^c^		HDRS^d^		YMRS^e^	
**Compliance first**																	
					*r*	1		0.77		–0.17	0.16	0.28		–0.08		–0.09	
					*P* value	—^f^		<.001		.32	.35	.35		.64		.58	
**Compliance total**																	
				*r*		0.77		1		–0.23	0.01	0.45		0.21		0.02	
				*P* value		<.001		—		.21	.96	.008		.25		.92	
**Age**																	
			*r*			–0.17		–0.23		1	–0.03	0.12		0.15		0.12	
			*P* value			.32		.21		—	.87	.49		.36		.47	
**Education**																	
		*r*				0.16		0.01		–0.03	1	0.08		–0.25		–0.27	
		*P* value				.35		.96		.87	—	.62		.12		.11	
**WASI IQ**																	
	*r*						0.28		0.45	0.12	0.08		1		0.07		0.05
	*P* value						.35		.008	.49	.62		—		.67		.79
**HDRS**																	
	*r*						–0.08		0.21	0.15	–0.25		0.07		1		0.58
	*P* value						.64		.25	.36	.12		.67		—		<.001
**YMRS**																	
	*r*						–0.09		0.02	0.12	–0.27		0.05		0.58		1
	*P* value						.58		.92	.47	.11		.79		<.001		—

^a^Compliance first: compliance rate for first 2 weeks of surveys.

^b^Compliance total: compliance rate for all 4 weeks of surveys.

^c^WASI IQ: Wechsler Abbreviated Scale of Intelligence.

^d^HDRS: Hamilton Depression Rating Scale score (at baseline).

^e^YMRS: Young Mania Rating Scale score (at baseline).

^f^Not applicable.

### Usability and Acceptability

Using the combined postassessment evaluation responses (Table 3), which 36 out of the 38 participants completed, 35 out of 36 participants (97%) did not have difficulties understanding the mood questions. While 15 out of 36 participants (42%) did not find the phone comfortable to carry, 33 out of the 36 participants (92%) did not have difficulties pressing response keys. When looking at evaluation responses for both BD and HC groups together, 34 out of 36 participants (94%) did not find that the daily questions took too much time to complete, and 27 out of 36 participants (75%) indicated that answering the questions was helpful for them to monitor their moods. In addition, 26 out of 36 participants (72%) indicated that participating in this study had been helpful/beneficial, and 32 out of 36 participants (89%) did not believe participation in our study took up too much time. Overall, 34 out of 36 participants (94%) believed this experience was pleasant, and 33 out of 36 participants (92%) would recommend others participate in a similar study.

**Table 3:** Subject ratings on poststudy evaluation for 36 out of 38 participants.

Evaluation question	Strongly agree/agree, n (%)	Neutral, n (%)	Strongly disagree/disagree, n (%)
I had difficulties understanding the survey questions.	0 (0)	1 (3)	35 (97)
Answering the questions was helpful for me to monitor my mood/functioning.	27 (75)	4 (11)	5 (14)
The daily tasks or use of the Jawbone activity tracker changed my behavior in some way.	21 (58)	5 (14)	10 (28)
Participation in this study took up too much time.	2 (6)	2 (6)	32 (89)
Participating in this study has been helpful/beneficial.	26 (72)	6 (17)	4 (11)
Overall, this experience was pleasant.	34 (94)	1 (3)	1 (3)
Overall, this experience was challenging.	13 (36)	3 (8)	20 (56)
I would recommend others participate in a similar study.	33 (92)	0 (0)	3 (8)

### Mixed Linear Effects Models

The multivariate mixed effect model for only the BD participants showed that an increase in text messages was associated with higher overall mood ratings (β=.04; *P*=.04). Decreased outgoing text messages was associated with an increase in rapid thoughts (β=–.02; *P*=.04). Total duration of calls was found to negatively associated with impulsivity ratings (impulsivity in thoughts and impulsivity in behavior) over time (thoughts: β=–.29; *P*=.02; behavior: β=–.16; *P*=.05).

## Discussion

### Principal Results

These results indicate that individuals who experience chronic and fluctuating changes in their mood are open to and respond adequately to twice-daily surveys about their mood using devices that they use on a regular basis, suggesting that this type of assessment could be acceptable to patients in practice. The completion rate of daily questions was similar to unaffected, healthy controls. Both diagnostic groups completed 91% of the questions sent to them. These findings suggest that this type of daily assessment is feasible to incorporate into measurement-based care initiatives and could provide a unique way of accurately and efficiently monitoring changes in functioning and clinical phenotypes. Further, our participants, including the individuals with BD, generally endorsed positive responses regarding use of the smartphone app. Using EMA on smartphones can serve as a way in which patients can self-monitor their symptoms and functioning, which subsequently could allow them to take part in the medical decision making. It could provide a quicker and cost-effective way to inform providers that patients are at risk or in the beginning stages of a relapse.

In line with our hypotheses, symptoms associated with increased energy and activation, including elevated mood, more impulsivity in thoughts and behaviors, and rapid thoughts, were associated with specific objective smartphone behaviors. Notably, higher mood was associated with more incoming text messages, rapid thoughts were associated with fewer outgoing text messages, and impulsivity was associated with shorter call duration. There was not one pattern of increased objective phone behavior associated with increased self-reported activity and activation states, but rather symptom-specific relationships. Our findings suggest that increasing mania-like symptoms correlate with decreasing phone communication habits using text messages and phone calls. The more mania-like symptoms, the less the participants engaged in communication activities on their phone, despite receiving more text messages. These are promising results that suggest that smartphones could be an easy and objective monitoring tool to capture illness activity in bipolar disorder, particularly shifts in mania-like symptoms. Further work will examine if passive phone behavior patterns can be predictive of future mood symptoms within individuals or are correlated with mania-like symptoms that meet threshold for a full manic episode. For example, can passive phone behaviors the week or two before a mood episode be predictive of the impending mood episode? Additional research could also investigate if other smartphone activities using sensor data (eg, accelerometer, access to social media, etc) also change with increasing mania symptoms.

We did not find a relationship between smartphone behaviors and depression-like symptoms, in contrast to our hypothesis and some recent literature [21], suggesting that it is the increase rather than decrease in psychomotor activity that is likely reflected in phone usage patterns. This may reflect how mood was captured, as depressive symptoms were only assessed via 1 question (eg, rate your mood from low to high). In contrast to Faurholt-Jepsen and colleagues [21], who found that greater depression symptoms were associated with more incoming calls, we did not find a relationship between mood and number of incoming or outgoing calls. Further, Faurholt-Jepsen and colleagues found that higher mood was associated with more outgoing texts and longer duration of calls, but they captured mood ranging from “depressive to manic” using a scale from –3 to +3, whereas we divided mania-like symptoms into some of mania’s component parts (rapid thoughts, impulsivity). We found that faster self-reported thoughts and greater impulsivity were associated with fewer outgoing texts and shorter call duration, respectively, indicating that mania-like symptomatology rather than global ratings of mood may have a differential effect on phone behaviors.

### Limitations

Mobile health studies of this kind, particularly as studies now are focusing on chronic mental health disorder, are in their infancy. Limitations may impede overall interpretation of findings and will need to be addressed in future studies. Given our small sample size, our group of participants with bipolar disorder may not be reflective of the broader, heterogeneous nature of the bipolar disorder illness. The design of our study was targeting a narrow group of individuals with bipolar disorder (those with frequent mood fluctuations who were generally highly educated) who are likely more technology oriented, motivated to participate in research, and more willing to complete daily assessments than is the larger bipolar disorder population. For example, our sample was already actively engaged in a longitudinal study of bipolar disorder, so they were likely more willing to engage in other research studies and participate in daily activities than are other individuals who are not volunteering their time. Frequencies regarding usability may therefore be skewed compared with the general bipolar disorder population. In general, our findings may not be generalized to the broader bipolar disorder population or general population who use phone apps. Along the same lines, we may lack adequate power to find other meaningful relationships in the data. These results are preliminary and may not be capturing the smartphone behaviors that are reflective of mood states, and further replication is needed. Future studies should examine the feasibility in measuring mood over longer study periods, as there may be a period when individuals no longer are motivated to or see any benefit in measuring their own mood over time. Further validation analyses are underway to examine the validation of our single mood items, as they relate to the gold standard, clinician-administered measures of mood. These results are limited by use of only Android system users, and this may be a different subgroup of individuals. There may also be constraints of having our participants use study-issued phones that may inadvertently interfere with their normal use of the phone. Lastly, we cannot state if a relationship exists between phone behaviors and subsequent emergence of a diagnosed manic episode because full mood episodes were not measured by a clinician rater.

### Conclusions

Overall, our sample of individuals diagnosed with bipolar disorder showed a very high adherence rate to the daily momentary assessments, and they generally had positive evaluation responses, indicating that our smartphone app was acceptable and that this type of study design is feasible to use in individuals with BD who have frequent fluctuations in mood. Use of this type of mobile technology shows promise as a way to collect important clinical information that can be used to inform treatment decision making and monitor outcomes in a manner that is not overly burdensome to the patient or providers. It further highlights the feasibility of collecting these types of data for measurement-based care for patients who are already engaged in mental health treatment. Our participant evaluations of the app indicated that they did not find this type of assessment burdensome and that it made them think about their mood and behavior. Future studies based on further development of this app are aimed at investigating if engaging patients to assess their own symptoms and daily functioning may empower self-awareness and self-management of symptoms and help patients identify triggers, track their own disease progression, and learn when to seek out care.

Smartphones, which are ubiquitous within our culture and used by the majority of individuals, including those with chronic conditions, hold immense opportunities to study human behavior. For those with bipolar disorder, smartphones and specifically communicative behavior on smartphones (eg, how often one makes phone calls, number of text messages sent and received) show promise by providing proxy information about illness activity in bipolar disorder. Changes in communicative behavior may indicate an increase in mania-like symptoms and further be an indicator for a problematic mood shift towards a manic episode. This type of research is in its infancy for mental health researchers, but studies of this kind can help launch future work to investigate the potential that technology-related behaviors have on understanding disease.

## Abbreviations

BD: bipolar disorder
DIGS: Diagnostic Interview for Genetic Studies
EMA: ecological momentary assessment
HC: healthy controls
